# Global, regional and national retinoblastoma burden in children under 10 years of age from 1990 to 2021: Trend analysis based on the Global Burden of Disease Study 2021

**DOI:** 10.1371/journal.pone.0327832

**Published:** 2025-07-08

**Authors:** SiQi Zhang, GuoXin Huang, Xiang Li, ZhiJie Zhang, KaiXin Peng, Lei Zhu, Chengda Zhang, Tong-tong Niu

**Affiliations:** 1 Xinjiang 474 Hospital, Urumqi, Xinjiang, China; 2 Department of Medical Oncology, Xiang’an Hospital of Xiamen University, School of Medicine, Xiamen University, Xiamen, Fujian, China; 3 Department of Evidence-Based Medicine Center, Xiangyang No.1 People’s Hospital, Hubei University of Medicine, Xiangyang, China; 4 Eye Institute and Affiliated Xiamen Eye Center, School of Medicine, Xiamen University, Xiamen, China; 5 Kunming Medical UniversityKunming, Yunnan, China; 6 Department of Hepatobiliary surgery, Sichuan Mianyang 404 Hospital, Mianyang, China; 7 North Sichuan Medical College, Nanchong, China; 8 Department of Clinical Laboratory, Xiangyang No.1 People’s Hospital, Hubei University of Medicine, Xiangyang, China; 9 Department of Neurosurgery, Xiangyang No. 1 People’s Hospital, Hubei University of Medicine, Xiangyang, China; All India Institute of Medical Sciences - Raipur, INDIA

## Abstract

**Background:**

Retinoblastoma (RB) is the most common malignant eye tumor in children, which poses a great threat to children’s vision and life. Comprehensive global, regional and country-level assessments of retinoblastoma in children under 10 years of age are important to help fine-tune health policies and rationalize the allocation of medical resources.

**Methods:**

Data on RB-related burden in children under 10 years of age were collected in the 2021 Global Burden of Disease (GBD) study to assess trends in RB burden using mean annual percentage change (AAPC). Absolute and relative health inequalities of RB burden were analyzed using slope index and concentration index. An age-period-cohort model was fitted using package NORDPRED to predict the future RB burden.

**Results:**

The global number of RB cases in children under 10 years of age in 2021 was 57,333 (95%UI: 34339.65,761.03), the annual standardized prevalence rate (ASPR) was 4.39(95%UI: 2.63, 5.95), and the AAPC (1990−2021) was 0.65(95%CI: 0.44, 0.86). Over the past 30 years, age-standardised mortality (ASMR) and age-standardised DALY(ASDR) have declined globally. At the level of socio-demographic index (SDI) regions, ASIR and ASPR were the highest in the medium-high SDI and high SDI regions, with ASPR being 6.03(95%UI: 3.01–9.21) and 5.44(95%UI: 3.97–7.18), and ASIR being 0.66(95%UI: 0.33–1.01) and 0.59(95%UI: 0.43–0.78). The mortality and DALYs of RB decreased gradually with the increase of SDI. At the country level, China and India are the countries with the highest number of cases, together accounting for about 30% of the global cases, and ASIR is still on the rise in these two countries. The inequality analysis shows that RB burden is heavier in countries with lower SDI. The number of RB cases worldwide is expected to rise slowly, but the global burden will gradually decrease.

**Conclusion:**

As one of the main causes affecting the life and health of children, with the increase in the number of diseases worldwide, it is necessary for decision-makers to customize relevant intervention policies to provide effective prevention and control measures to help achieve the global Sustainable Development Goals.

## Introduction

Retinoblastoma (RB) is the most common ocular malignancy in children, with 80% diagnosed before the age of 3 [1]. If not treated promptly, it can lead to death [2]. The global disease burden of RB has become an important factor in children’s health. There are 9000 new cases worldwide every year, and 80% of cases occur in low-and middle-income countries, which also have the highest mortality rates [3]. Mortality rates for children with RB are reported to be 40% to 70% in Asia and Africa, compared with only 3% to 5% in some high-income countries [4], which is associated with delayed diagnosis and not receiving appropriate treatment [3,5]. Therefore, a range of health interventions can be undertaken to develop standard RB management protocols, thereby improving global RB cure and rescue rates [6]. In addition, understanding the relative contributions of RB risk factors to RB GBD and trends in RB risk factors is critical to inform global RB control efforts.

Most studies on RB have focused on pathogenesis and treatment options [7–9]. Epidemiological studies on RB have been limited to regions or individual countries [10,11]. No studies have focused on the global and regional disease burden of RB in children under 10 years of age. Large-scale epidemiological studies can shed new light on the disease burden of RB on human health, and provide reliable evidence for improving public health and rational allocation of medical resources [6]. The GBD database provides an opportunity for in-depth analysis and prediction of global disease burden.

In the Global Burden of Diseases, Injuries, and Risk Factors (GBD) study, which systematically quantified the health burden of 371 diseases and injuries by age, sex, year, and geographic location [12,13]. We describe the latest epidemiology of RB in children under 10 years of age for the first time, analyzing global, regional, and national incidence, prevalence, mortality, and disability-adjusted life years (DALY) of RB and projections for RB from 1990 to 2021.

## Materials and methods

### Data sources

Data from the GBD 2021 study, available through the Global Health Data Exchange (https://ghdx.healthdata.org/gbd-2021/sources) [13], were used to quantify the global burden of RB in children under 10 years of age by comprehensively assessing the prevalence, incidence, mortality, and DALY of RB. Statistical analyses were also performed for different social-demographic index (SDI) regions. WHO defines a child as under 10 years of age. We collected gender data on RB from the age group under 10 years and grouped them by 21 regions or countries defined by GBD that were geographically close and epidemiologically similar. In the GBD2021 study, according to the tenth edition of the International Classification of Diseases (ICD-10), the code for retinoblastoma is C69.2-C69.22. GBD2021 classifies causes into four levels, with retinoblastoma classified as a grade 4 cause [13–15].

Prior to analysis, we performed standard data cleaning procedures, including consistency checks for duplicated entries, logical validation (e.g., incidence should not exceed prevalence), and range verification to ensure all values fell within expected bounds (based on GBD documentation). Missing values were minimal due to the modeling framework used by GBD, which imputes data using DisMod-MR 2.1 and cause-of-death Integration Models (CODEm) when primary data are unavailable. Moreover, we visually inspected time-series plots to detect anomalous trends and confirmed the plausibility of imputed values using metadata from GBD technical appendices.

All modeling processes and data sources used in GBD 2021 have been extensively peer-reviewed and are publicly documented. Full methodological details are available in the GBD 2021 capstone papers and the online GBD methodology repository.

### Sociodemographic index

The Sociodemographic Index (SDI) is a comprehensive indicator introduced by the Institute for Health Metrics and Evaluation (IHME) in 2015 to assess the development of a country or other geographical region [16]. The evaluation indicators are per capita income, education and total fertility [17]. On a 0–1 scale, the SDI quintile corresponds to five regions: low, medium-low, medium, medium-high, and high [13]. At the same time, SDI is dynamic, that is, SDI is divided by year and country, and the SDI classification of some countries or regions may change over time.

### Disease burden indicator

The GBD study took all available population-representative studies, large-scale surveys, hospital records, etc., to categorize the incidence and prevalence of each disease [18,19]. For most diseases and injuries, GBD2021 models incidence and prevalence using DisMod-MR 2.1, a Bayesian disease modeling meta-regression tool that generates internally consistent estimates of prevalence, morbidity, and mortality by sex, location, year, and age group [13,20]. DisMod-MR 2.1 also predicts the incidence and prevalence of missing sites. Cause-of-death estimates for most diseases and injuries are modeled using cause-of-death Integration Models (CODEm), a modeling tool developed specifically for GBD to evaluate the validity of out-of-sample predictions for different statistical models and covariate permutations, and then combine the results of these assessments to generate cause-specific burden of death estimates [14,21]. It can estimate the number of deaths from a given cause by location, age, sex, and year. DALYs refers to the years of healthy life lost from disease onset to death, including years of life lost (YLL) and years of disabled life (YLD) calculated by year, age, sex and region [14,20]. DALYs is an important index to quantify the burden of disease.

### Statistic analysis

In line with the GBD methodology, the estimates are presented with 95% uncertainty intervals (UIs), which reflect the range of values derived from the modeling process. Unlike traditional confidence intervals (CIs), UIs account for uncertainty from various sources including model input data, statistical estimation methods, and parameter assumptions.

In this study, we quantified the prevalence, incidence, mortality, and DALY of RB in children under 10 years of age using age-standardized rates (ASR) and their 95% UI. Predictors per 100,000 population, including 95% of UI calculated using the world standard population, were estimated using Joinpoint regression models for global and regional age-standardized incidence rate(ASIR), age-standardized prevalence rate(ASPR), Time trends in age-standardized mortality rate(ASMR) and age-standardized DALY rate(ASDR). This model is commonly used in epidemiological studies to identify and characterize significant points of change in RB time series at global and country scales [22,23]. Average annual percentage change (AAPC) was calculated during the study period 1990–2021 to assess overall trends.AAPC estimates with a 95% CI lower bound greater than zero indicate an upward trend within a specified interval, The estimate plus the 95% UI upper bound less than zero indicates a downward trend, and the trend is stable when the 95% UI of AAPC contains zero [22].

To achieve universal health and inform policies, planning and practices to reduce health inequalities, we performed absolute and relative health inequality analyses for RB using the Slope Index (SII) and the Concentration Index (CI), respectively [24,25]. The Slope Index was calculated by regressing each country’s DALYs on the SDI-related Relative Position Scale, defined by the midpoint of the cumulative population range of SDI rankings. The Concentration Index was calculated by calculating the ratio of the area between the Lorentz curve and the diagonal, The curve was fitted using cumulative SDI rank and cumulative DALY scores [26]. Global incidence, prevalence, mortality, and DALY were also predicted to 2040 to assess future GBD of RB. The prediction was based primarily on global population projections for 2017–2100 and age-normalized RB incidence, prevalence, mortality, and DALY data for 1990–2021. An age-period-cohort model was fitted to recent trends. The model is implemented in R via the package NORDPRED [27–29]. All statistical analysis and visualizations are processed using R software, *P* < 0.05 was considered statistically significant.

## Results

### Disease burden globally and in 5 SDI regions

The number of RB cases in children under 10 years of age worldwide in 2021 is 57,333 (95%UI: 34339.65,761.03) cases, age-standardized prevalence rate (ASPR) was 4.39 (95%UI: 2.63, 5.95)/100000, an increase of 0.88/100000 compared to 1990, AAPC (1990−2021) is 0.65 (95% CI: 0.44, 0.86). Similarly, the age-standardized incidence rate (ASIR) of RB increased from 0.39(95%UI: 0.25, 0.50)/100000 in 1990 rose to 0.48(95%UI: 0.29, 0.65)/100000 in 2021, AAPC is 0.64 (95%CI: 0.43, 0.85). Notably, the age-standardized mortality rate (ASMR) for RB declined globally, from 0.26(95%UI: 0.15, 0.35) in 1990 decreased to 0.21(95%UI: 0.13, 0.29) in 2021, and AAPC is −0.68 (95%CI:-0.84,-0.52). Meanwhile, the age-standardized DALYs rate(ASDR) of global RB also showed a downward trend, being 18.67(95%UI: 11.30, 25.61) in 2021 (**Table 1**).

**Table1:** Global prevalence, morbidity, mortality, and DALYs of RB in 1990 and 2021, and trends from 1990 to 2021.

	Incidence (95%UI)			Prevalence (95%UI)			Deaths (95%UI)			DALYs (95%UI)		
	ASR per 100000(1990)	ASR per 100000(2021)	AAPC (1990-2021)	ASR per 100000(1990)	ASR per 100000(2021)	AAPC (1990-2021)	ASR per 100000(1990)	ASR per 100000(2021)	AAPC (1990-2021)	ASR per 100000(1990)	ASR per 100000(2021)	AAPC (1990-2021)
Global	0.39(0.25-0.50)	0.48(0.29-0.65)	0.64 (0.43 to 0.85)	3.51(2.27-4.54)	4.39(2.63-5.95)	0.65 (0.44 to 0.86)	0.26(0.15-0.35)	0.21(0.13-0.29)	−0.68 (−0.84 to −0.52)	23.00 (12.97-30.40)	18.67 (11.30-25.61)	−0.67 (−0.82 to −0.51)
Low SDI	0.65(0.36-0.90)	0.52(0.29-0.78)	−0.68 (−0.81 to −0.55)	5.87(3.25-8.18)	4.72(2.68-7.10)	−0.66 (−0.8 to −0.53)	0.65(0.36-0.90)	0.45(0.27-0.66)	−1.14 (−1.3 to −0.98)	56.69 (31.48-78.80)	39.38 (23.58-57.45)	−1.14 (−1.3 to −0.98)
Low-middle SDI	0.35(0.19-0.50)	0.40(0.23-0.61)	0.48 (0.32 to 0.63)	3.22(1.77-4.56)	3.69(2.06-5.53)	0.49 (0.34 to 0.64)	0.34(0.18-0.48)	0.24(0.15-0.35)	−1.01 (−1.23 to −0.8)	29.30 (15.75-41.95)	21.38 (12.80-30.32)	−1.01 (−1.22 to −0.79)
Middle SDI	0.23(0.13-0.35)	0.42(0.22-0.61)	1.81 (1.54 to 2.08)	2.10(1.19-3.15)	3.82(2.03-5.58)	1.82 (1.55 to 2.09)	0.17(0.10-0.22)	0.09(0.05-0.12)	−2.15 (−2.38 to −1.92)	14.49 (8.43-19.44)	7.73 (4.37-10.57)	−2.09 (−2.32 to −1.87)
High-middle SDI	0.33(0.22-0.51)	0.66(0.33-1.01)	1.87 (1.26 to 2.48)	3.03(1.97-4.62)	6.03(3.01-9.21)	2.05 (1.24 to 2.87)	0.12(0.07-0.18)	0.04(0.02-0.06)	−3.45 (−3.65 to −3.24)	10.34 (5.77-16.12)	3.97 (1.95-5.96)	−3.2 (−3.41 to −2.99)
High SDI	0.67(0.52-0.84)	0.59(0.43-0.78)	−0.27 (−0.77 to 0.23)	6.11(4.77-7.68)	5.44(3.97-7.18)	−0.27 (−0.77 to 0.23)	0.03(0.03-0.04)	0.01(0.01-0.01)	−3.44 (−4.22 to −2.66)	3.19 (2.62-3.86)	1.32 (1.03-1.66)	−2.9 (−3.89 to −1.9)

Abbreviations: SDI: Socio-Demographic Index; AAPC:Average annual percentage change; DALYs: Disability-adjusted life years.

At SDI regional level, ASPR and ASIR of RB in 2021 are highest in the medium – high and high SDI regions,ASPR is 6.03(95% UI: 3.01–9.21) and 5.44 (95%UI: 3.97, 7.18) respectively. ASIR is 0.66(95%UI: 0.33–1.01) and 0.59(95%UI: 0.43, 0.78) respectively. However, ASPR and ASIR in low SDI and high SDI regions showed a downward trend compared with 1990. It is noteworthy that mortality and DALYs in RB decreased gradually with increasing SDI, and ASMR and ASDR in all five SDI regions showed a downward trend from 1990 to 2021 (**Table 1**).

### Burden of disease in 21 GBD regions

**Fig 1:**
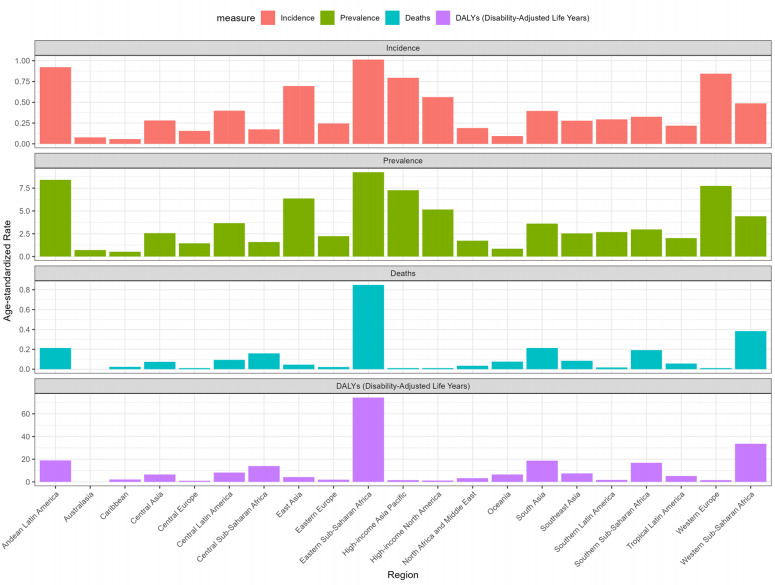
Age-standardised incidence, age-standardised prevalence, age-standardised mortality, and age-standardised DALYs for 21 GBD regions in 2021.

Eastern Sub-Saharan Africa has the heaviest RB disease burden in 2021, with ASIR: 1.01(95%UI: 0.61, 1.6), ASPR: 9.24(95%UI: 5.59, 14.58), ASMR: 0.85(95%UI: 0.55, 1.3) and ASDR: 74.26(95%UI: 48.07, 113.3) ranking first in the world in 2021 (Fig 1). In contrast,Western Europe, High-income Asia Pacific, East Asia and High-income North America also have the highest ASIR and ASPR in the world, but the ASMR and ASDR of these regions are much lower than those of Eastern Sub-Saharan Africa, indicating that the economic conditions and medical conditions of these regions are better than those of Eastern Sub-Saharan Africa. In addition, the ASPR of East Asia in 2021 is 6.36(95%UI: 2.42, 10.15) and ASIR 0.69(95%UI: 0.26, 1.11) Compared with 1990, there is a significant increase, AAPC is 3.11(95%CI: 2.52, 3.7) and 3.1(95%CI: 2.51, 3.69). However, ASMR and ASDR in East Asia showed a downward trend. In the past 30 years, the burden of RB decreased most significantly in Australia, where the prevalence, incidence, mortality and DALY of RB all showed a downward trend (S1 and S2 Tables).

### National disease burden in 2021

**Fig 2:**
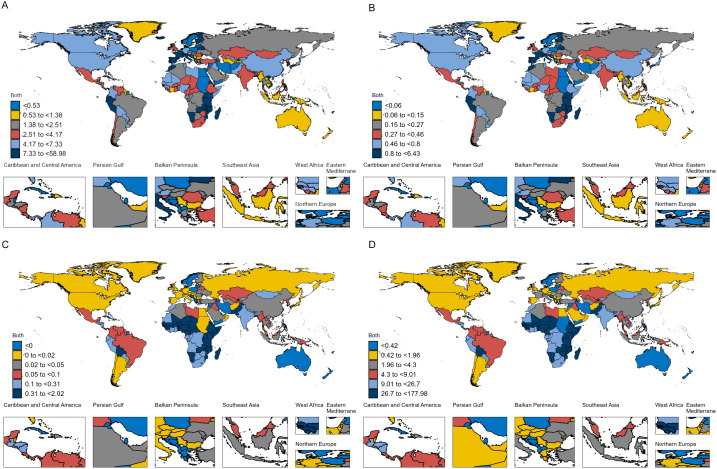
RB burden for 204 countries or regions worldwide in 2021. A: ASPR, B:ASIR, C:ASMR, D:ASDR. In 2021, five countries had RB in children under 10 years of age ASMR exceeding 1/100000, namely Malawi, Tokelau, Kenya, Eritrea and Mozambique. From 1990 to 2021, we observed that Tokelau was the country with the largest increase, with AAPC reaching 15.43 (95%CI: 11.33, 19.67). In addition, Tokelau, Malawi, Kenya, Eritrea and Mozambique have the highest ASDR of RB in 2021, among which the ASDR of the first three countries exceeds 100/100000, respectively 177.98.(95%UI: 36.27,587.69),177.15 (95%UI: 69.62,410.87),159.78 (95%UI: 89.24,259.67), and Tokelau is still the country with the largest increase. It can be seen that the burden of RB in Tokelau is on the rise.

At the national level, the burden of RB in children under 10 years of age varies greatly among countries in the world in 2021 (Fig 2). The country with the highest number of cases is China 10091 (95% UI: 3770.79, 16239.44), followed by India 7395 (95%UI: 3921.16,11824.07), Nigeria3584 (95%UI: 1177.72,6597.77), Kenya2768 (95%UI: 1412.66,4637.89), Pakistan2523 (95%UI: 1019.46,4871.84). ASIR from <0.001 for Seychelles to 6.43(95%UI: 1.36, 20.83) for Tokeau. Of the 204 countries, 23 countries had RB ASIRs greater than 1/100000 and 55 countries had ASIRs less than 0.1/100000. Similarly, Tokeau had the highest ASPR(58.98/100000), followed by Kenya 23.49 (95%UI: 12.00,39.36), Portugal20.50 (95%UI: 12.41,25.41), Malawi20.15 (95%UI: 7.62,43.65), Finland17.15 (95%UI: 9.33, 24.01) and Switzerland 16.47 (95%UI: 8.01, 2.74). From 1990 to 2021, Tokelau also had the largest increase in ASPR and ASIR, AAPC is 19.29(95%UI: 15.31, 23.41), followed by Armenia, Cook Islands, Georgia, Niue and Guyana(S3 and S4 Tables).

### Inequality analysis

**Fig 3:**
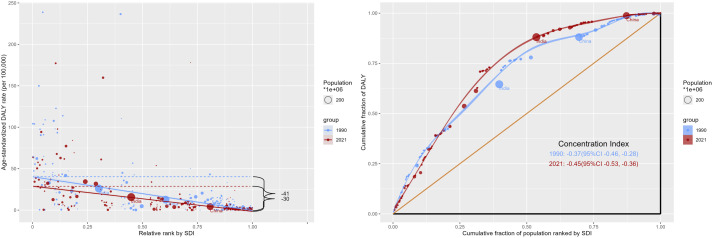
Health inequality Slope Index and Concentration Index for the DALYs of RB from 1990 to 2019 across the world.

By analyzing the inequality of SDI-related RB burden, the slope index shows that the difference in DALYs ratio between the countries with the highest SDI and the countries with the lowest SDI in 1990 was 40.81 (95%CI: 36.04, 45.58), the gap decreased to 30.32(95%CI: 27.18, 33.47) in 2021. The concentration index increased from −0.37 (95%CI-0.46,-0.28) in 1990 to −0.45 (95%CI-0.53,-0.36) in 2021 (**Fig 3**). The results show that the absolute and relative inequality of RB burden is higher for countries with lower SDI.

### GBD Future Forecast for Global RB

**Fig 4:**
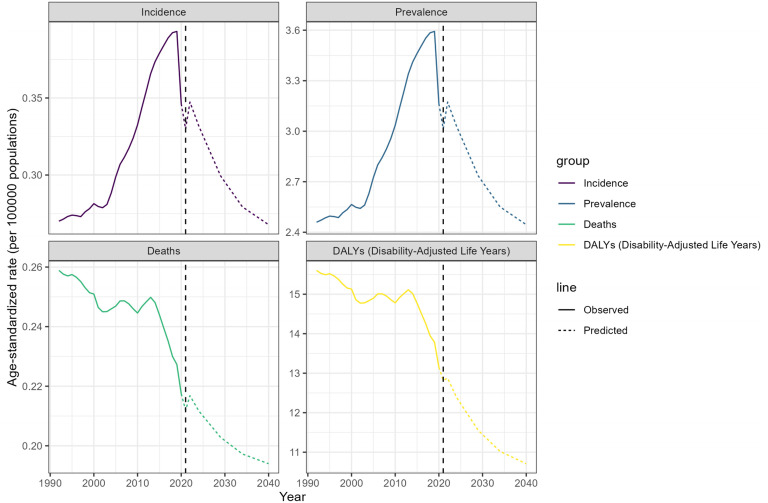
The future forecasts of GBD in RB.

(**Fig 4**) Shows the future prediction of RB. It is expected that the ASIR, ASPR, ASMR and ASDR of RB will gradually decrease, that is, the disease burden of RB is expected to decrease. The number of RB cases is expected to be 43781 in 2040, and the ASPR is 3.56/100000, which is 18.9% lower than that in 2021. We also estimate the ASIR, ASMR and ASDR of RB in the world from 2021 to 2040, and the ASIR of RB is expected to be 0.39/100000 in 2040, 18.75% lower than 2021. ASMR was 0.19/100000, 8.5% lower than 2021, ASDR was 15.6/100000, 16.44% lower than 2021.

## Discussion

Retinoblastoma is the most common eye cancer in children, and it is an important challenge for global public health. Timely and effective treatment can save the survival and vision of patients [30]. Because it is the first cancer with genetic causes to attract people’s attention, its etiology has been well understood, and the treatment mode of RB has gradually been innovated [2]. However, due to insufficient public and medical awareness, in some low-income countries, The mortality rate of retinoblastoma remains high [3]. There is a lack of studies on the prevalence, incidence, mortality, and DALYs of RB in children under 10 years of age in different countries and regions worldwide. Our analysis included 204 countries worldwide and spanned 30 years to detail the global disease burden of RB. This study innovatively assesses time trends in RB burden and makes projections for future disease burden of RB.

Our analysis estimates the global prevalence of RB under age 10 to be 57333 in 2021, with an age-standardized prevalence of 4.39 (95%UI: 2.63, 5.95)/100000,RB is a rare malignant tumor, but because it is most common in childhood, the harm of RB should not be underestimated. From 1990 to 2021, the incidence and prevalence of RB in the world gradually increased, while the mortality and DALY gradually decreased. This indicates that with the advancement of RB research,RB treatment has been well developed. The improvement of treatment strategies has led to a gradual increase in the cure rate and salvage rate of RB, but the incidence and prevalence of RB are on the rise, which means that the number of RB patients will continue to increase. This trend can be attributed to the progress made in the diagnosis, treatment and prognosis of RB in the health care system, so that the early diagnosis and timely and effective treatment of RB patients are guaranteed [2].

This increase may be partly due to improved diagnostic capacity, more robust reporting systems, and increased awareness among health professionals and parents. In addition, global population growth and the resulting larger number of children under 10 years of age may contribute to the absolute burden. Enhanced survival rates of RB patients due to treatment improvements may also raise the number of prevalent cases. Moreover, the continuous improvement of GBD modeling techniques and the integration of additional data sources over time could also influence prevalence estimates.

The simultaneous increase in RB prevalence and decrease in DALY may appear contradictory but is consistent with the improving diagnosis, treatment, and long-term survival of RB patients. As more cases are identified and treated effectively—especially in countries with developing healthcare infrastructure—more children live with RB, contributing to higher prevalence. At the same time, advances in treatment, such as targeted chemotherapy and earlier diagnosis, reduce both mortality (YLL) and disability (YLD), leading to an overall decline in DALYs. Thus, the increasing prevalence reflects greater case recognition and survival, whereas the falling DALY reflects reduced severity and lethality of the disease.

Compared with 1990, the RB burden in high SDI areas decreased gradually, indicating that the prevalence of RB in areas with better economic conditions and medical conditions has improved, and effective prevention and treatment measures have been improved. This is consistent with the results of previous studies [31]. The results of single-center studies in many developed countries show that the survival rate of RB is above 95%. Even 100% in the UK [32–34]. Single-center studies in low-income countries showed RB5-year survival rates of 60% or less, and in Nepal 10-year survival rates of only 24% [35–37]. And most deaths in these low-income countries were metastatic. A cross-sectional study indicates that lower regional income levels lead to a higher age of diagnosis of RB, resulting in an increase in locally advanced and distant metastatic cases [6]. Therefore, the survival rate of RB patients varies greatly in different development level regions, which is related to the differences in RB management choices among countries.

In different geographical regions and countries, there are also large differences in their RB disease burden. Eastern Sub-Saharan Africa has the highest RB disease burden among 21 GBD regions, probably due to the lack of medical resources in the region and the lack of early diagnosis and timely treatment of children. Compared with other regions with high prevalence and incidence, such as Western Europe, High-income Asia Pacific, East Asia and High-income North America, The prevalence of neonatal screening and preventive treatment in these areas has resulted in low mortality and DALY in these areas. At the national level, China and India are the countries with the highest number of cases, accounting for approximately 30% of all cases worldwide, and the ASIR in these two countries is still on the rise, with AAPC of 3.1(95% CI: 2.49, 3.7) and 0.7(95%CI: 0.4, 1.01), but it is encouraging that ASMR and ASDR are on a downward trend due to continued innovation in RB treatment modalities, including advances in surgery and chemotherapy regimens [2]. Positive change is imminent, as genomic science and global communications enable all children and families affected by retinoblastoma to have an equal chance of cure, and regulators must make policy optimization for scientific research and building health system capacity.

Through inequality analysis, we know that global RB has a higher disease burden in regions with low SDI. It is vital that retinoblastoma is treated at an early stage. Delayed diagnosis and treatment of RB in medium-low income countries may play a role in this phenomenon [38,39]. This study suggests that there is an urgent need to improve the diagnosis and treatment of RB in medium-low income countries, and that relevant authorities need to take measures to address this challenge in conjunction with more resourced national centers.RB cases are approximately 9000 per year, Although rare compared to common tumors, the curable rate of RB makes it achievable to take a range of actions to make RB a zero-mortality malignancy [40]. Our predictive analysis also suggests that the disease burden of RB will gradually decrease in the future, but this requires concerted action by countries around the world to improve the diagnosis and treatment of RB [6]. Learn lessons about retinoblastoma management and ensure that all children with retinoblastoma receive optimal lifesaving and vision care goals. Greatly improve the chances of survival and quality of life of children with retinoblastoma.

There are limitations to our study. First, the data we used in the GBD2021 study were collected from different sources. Although the GBD database is constantly updated and expanded, it is not possible to obtain data from all countries and regions in the world, so the completeness and timeliness of the data may be problematic, especially in some low SDI regions, and thus may lead to bias. Second, differences in disease management between different countries and regions may affect comparability. Finally, Limited by the sparsity of the data at potentially burdensome locations, combined with strong geographic trends, this creates the possibility for location-based modeling to underestimate geographic gradients.

## Conclusion

In summary, our study, based on the GBD2021 study, reveals the incidence, prevalence, mortality, and DALYs of RB in children under 10 years of age worldwide, and analyzes trends from 1990 to 2021 to determine the global disease burden of RB and predict the burden of RB in the next 20 years. This helps to improve the disease burden of the most common eye cancer in children worldwide and has important implications for preventing and controlling RB harm to children.

## Supporting information

S1 Table Incidence rate, morbidity, mortality and DALY of retinoblastoma in children under 10 years of age in 21 GBD regions and 5 SDI regions in the world from 1990 to 2021. (XLSX)

S2 Table AAPC of retinoblastoma in children under 10 years of age in 21 GBD regions and 5 SDI regions in the world from 1990 to 2021. (XLSX)

S3 Table Incidence rate, morbidity, mortality and DALY of retinoblastoma in children under 10 years of age in 204 countries from 1990 to 2021. (XLSX)

S4 Table AAPC of retinoblastoma in children under 10 years of age in 204 countries from 1990 to 2021. (XLSX)
